# Using Existing Clinical Data to Measure Older Adult Inpatients’ Frailty at Admission and Discharge: Hospital Patient Register Study

**DOI:** 10.2196/54839

**Published:** 2024-10-28

**Authors:** Boris Wernli, Henk Verloo, Armin von Gunten, Filipa Pereira

**Affiliations:** 1 Swiss Centre of Expertise in the Social Sciences (FORS) Lausanne Switzerland; 2 University of Applied Sciences and Arts Western Switzerland (HES-SO) Sion Switzerland; 3 Service of Old Age Psychiatry Lausanne University Hospital Lausanne Switzerland

**Keywords:** frailty, frailty assessment, electronic patient records, functional independence measure, routinely collected data, hospital register, patient records, medical records, clinical data, older adults, cluster analysis, hierarchical clustering

## Abstract

**Background:**

Frailty is a widespread geriatric syndrome among older adults, including hospitalized older inpatients. Some countries use electronic frailty measurement tools to identify frailty at the primary care level, but this method has rarely been investigated during hospitalization in acute care hospitals. An electronic frailty measurement instrument based on population-based hospital electronic health records could effectively detect frailty, frailty-related problems, and complications as well be a clinical alert. Identifying frailty among older adults using existing patient health data would greatly aid the management and support of frailty identification and could provide a valuable public health instrument without additional costs.

**Objective:**

We aim to explore a data-driven frailty measurement instrument for older adult inpatients using data routinely collected at hospital admission and discharge.

**Methods:**

A retrospective electronic patient register study included inpatients aged ≥65 years admitted to and discharged from a public hospital between 2015 and 2017. A dataset of 53,690 hospitalizations was used to customize this data-driven frailty measurement instrument inspired by the Edmonton Frailty Scale developed by Rolfson et al. A 2-step hierarchical cluster procedure was applied to compute e-Frail-CH (Switzerland) scores at hospital admission and discharge. Prevalence, central tendency, comparative, and validation statistics were computed.

**Results:**

Mean patient age at admission was 78.4 (SD 7.9) years, with more women admitted (28,018/53,690, 52.18%) than men (25,672/53,690, 47.81%). Our 2-step hierarchical clustering approach computed 46,743 inputs of hospital admissions and 47,361 for discharges. Clustering solutions scored from 0.5 to 0.8 on a scale from 0 to 1. Patients considered frail comprised 42.02% (n=19,643) of admissions and 48.23% (n=22,845) of discharges. Within e-Frail-CH’s 0-12 range, a score ≥6 indicated frailty. We found a statistically significant mean e-Frail-CH score change between hospital admission (5.3, SD 2.6) and discharge (5.75, SD 2.7; *P*<.001). Sensitivity and specificity cut point values were 0.82 and 0.88, respectively. The area under the receiver operating characteristic curve was 0.85. Comparing the e-Frail-CH instrument to the existing Functional Independence Measure (FIM) instrument, FIM scores indicating severe dependence equated to e-Frail-CH scores of ≥9, with a sensitivity and specificity of 0.97 and 0.88, respectively. The area under the receiver operating characteristic curve was 0.92. There was a strong negative association between e-Frail-CH scores at hospital discharge and FIM scores (r_s_=–0.844; *P*<.001).

**Conclusions:**

An electronic frailty measurement instrument was constructed and validated using patient data routinely collected during hospitalization, especially at admission and discharge. The mean e-Frail-CH score was higher at discharge than at admission. The routine calculation of e-Frail-CH scores during hospitalization could provide very useful clinical alerts on the health trajectories of older adults and help select interventions for preventing or mitigating frailty.

## Introduction

Switzerland’s current declared health care policy and its overall national system both aim to support older adults who wish to age healthily in their own homes. Recent statistical trends show increasing numbers of older adults who are multimorbid being hospitalized, thus putting age-related diseases and chronic health conditions at the forefront of the many concerns facing acute health care systems [1,2]. There is growing evidence that frailty syndrome may be a relevant acute hospital care clinical alert to predict complications associated with adverse outcomes [3-6]. Although there is a consensus on the definition of frailty—but perhaps not always on how to measure it—it is widely considered to be a dynamic geriatric condition characterized by an increased vulnerability to external dysfunction, a complex etiology, and intrinsic difficulties distinguishing it from normal aging [7,8]. Frailty is a preclinical state not directly associated with any disease or disability. The scientific literature documents a phenotypical approach to identifying frailty. It uses 3 or more of Fried 5 criteria (unintentional weight loss, exhaustion, low energy expenditure, slowness, and weakness) to recognize prefrailty and the deficit accumulation approach based on Rockwood Clinical Frailty Scale engineering theory [9]. In the phenotypical approach, 3 or more of Fried 5 criteria (unintentional weight loss, exhaustion, low energy expenditure, slowness, and weakness) are used to recognize prefrailty. In Rockwood deficit accumulation approach, frailty is associated with known comorbidities and disabilities, polypharmacy, and the relative risks of adverse drug reactions, rehospitalization, health services use, age-associated sensory deficits, and a lack of social support [9].

Routine hospitalization data stored in patients’ electronic health records (EHRs), including social, clinical, medical, and pharmacy data, could be important sources of frailty detection information during hospitalization and for assessing and comparing the different phases of hospitalization, such as in admission and discharge [10,11]. However, EHRs do not currently enable the construction of a standard reference measure of frailty for clinical and research purposes—they require customization. Researchers would require different data types (eg, diagnoses, clinical data, and health service codes) collected over specified periods using specific, discriminating coding methods [10,12]. Indeed, the length of the assessment period during which codes were measured could affect the measure’s accuracy when capturing certain chronic conditions. Another challenge is that coding systems and medical practice can change over time or across geographical regions. Switzerland’s different regional health care systems all use the *ICD-10* (*International Classification of Diseases, Tenth Revision*), as their medical coding system [13]. New billing codes are generated for new health care procedures (in the *ICD-11* [*International Classification of Diseases, Eleventh Revision*]) and services (eg, SARS-CoV-2 vaccination), and numerous codes are withdrawn each year [14]. Therefore, how well the frailty measures routinely collected by health care professionals perform should be evaluated periodically using updated datasets [15]. Developing a frailty measure from patients’ EHRs requires restricting the population to individuals with high rates of data completeness within the system to avoid bias due to missing data [16]. Few studies have explored customizing database-derived frailty measures using EHRs from a hospital register [10,12,16,17]. Measures using EHRs focus on investigating exposure to the risks of a particular outcome (such as syndromes, death, or reoccurrences of a disease) based on the selection of a group of patient characteristics and using a statistical model to explore the effectiveness of interventions and treatments among hospitalized inpatients [18]. However, any model’s usefulness and relevance depend on its accuracy. Few studies have investigated the robustness of frailty measures taken from routinely collected datasets using 2 approaches to frailty simultaneously [10]. Indeed, the Fried phenotypic approach has found a correlation of 0.6-0.7 between hospital EHRs and retrospective medical assurance data; the Rockwood deficit-accumulation approach has found correlation coefficients of 0.2-0.6 [10]. In particular, a recent study by Kim et al [19] showed that a frailty index, estimated using data routinely collected by health care professionals for older adults’ medical assurance claims (equivalent to Medicare claims), performed better than a comorbidity index for predicting disability, mobility impairments, recurrent falls, and days spent in a skilled nursing facility [19]. However, an e-Frailty Index >0.19, a threshold for frailty developed in the United Kingdom, had a positive predictive value of 0.11 (*R*^2^) for death in the next 3 months among primary care patients in that country [19]. Nevertheless, a database-derived measure of frailty might be able to provide a clinical alert of frailty across older adults, although it would be less readily interpretable deterministically for a particular individual. A cutoff point for positive screening can be determined according to percentile distributions (eg, top 5%), to sensitivity and specificity to a state of frailty (eg, 90% sensitivity for detecting a frailty phenotype), or to predefined, clinically relevant thresholds (eg, ≥0.2 according to a deficit accumulation frailty index) after considering clinical contexts (eg, inpatient or preoperative screening) and the resources available for detailed assessments and care management [20].

Few studies to date have used a database-derived frailty measurement score as a clinical alert (ie, change in frailty levels over time) to estimate the effects of an intervention (medical and medication treatment) or hospitalization (length of stay). In addition, frailty measures’ responsiveness to improvements or deteriorations in health status is still under investigation, as is the minimum clinically significant change they can recognize. Most of the research done in North America, Western Europe, and Asia has explored measures of frailty developed from primary care EHRs [10,11,21,22]. The items of clinical knowledge selected for these measures used data relevant to frailty’s overall clinical picture [23]. This paper’s overall aim is to demonstrate that data from hospital registers, which collect clinical and administrative information (for billing), can be used to identify a clinical alert of frailty among patients at the time of their hospital discharge. We hypothesized that the e-CH-frailty (Switzerland) clinical alert would significantly increase hospital discharges compared to the e-CH-frailty scores at hospital admission.

## Methods

### Study Design

We conducted a register-based study of patients’ routinely collected EHR data to customize a database-derived frailty measure. Data came from a large, longitudinal EHR dataset extracted from the register of a multisite public teaching hospital in Switzerland [24]. Once a database-derived frailty measure is developed, the critical next step is validating it against a reference standard, 1D, or multidimensional measure of frailty [20]. Due to the lack of any routine frailty assessment tool within our database, we selected the multidimensional Functional Independence Measure (FIM) [25], recommended by Dodds et al [25], Carlson et al [26], and De Saint-Hubert et al [27], as an alternative means of convergent validation [19,20].

### Study Population and Variables

The dataset comprised the Valais Hospitals’ sociodemographic, clinical, medical, and drug data. Valais Hospitals is a multisite public hospital in Switzerland serving a population of almost 360,000. It recorded over 40,000 hospitalizations and 650,000 ambulatory visits in 2023, mainly at its 2 primary hospital centers, 1 in each of the canton’s 2 distinct linguistic regions [25]. The EHR dataset included all inpatients aged 65 years or older admitted or readmitted between January 1, 2015, and December 31, 2017 (N=53,690). Incomplete records with more than 20% missing health data or records without sociodemographic data were excluded (excluded records numbered 6947, 12.93%). This study did not consider older adult inpatients who were admitted to the emergency department but returned to their homes within 24 hours. These 3 years were selected based on the availability of systematic, well-coded patient data. Without unique patient identifiers, the number of different patients and their readmission rates could not be explored. Per this study’s aims, we included the sociodemographic variables of age, gender, prehospital provenance, and discharge destination or death during hospitalization (Table 1). For patients’ physical status, we included the variables of general mobility, mobility for changing position, gait, balance disorders, fall risk, exhaustion, independence in upper- and lower-body care, upper- and lower-body dressing or undressing, and bladder continence (Multimedia Appendix 1). Unfortunately, the EHR did not include data on the older adult inpatients’ nutritional status for the selected period (January 2015 to December 2017). For their cognitive status, we included the variables of alertness or consciousness, orientation, concentration, verbal expression, capacity and skills to react to the demands of daily life, and ability to learn (Table 2). In addition, we noted the number of comorbidities calculated at each hospitalization (using *ICD-10*) [28].

**Table 1:** The sociodemographic data used to construct the e-Frail-CH^a^ measure (N=53,690).

Variables			Total (%)
**Gender, n (%)**			
	Men	25,672 (47.81)	
	Women	28,018 (52.18)	
**Age (years)**			
	Mean (SD)	78.37 (7.91)	
	Median (IQR)	78 (72-84)	
	Minimum-maximum	65-106	
**Age categories (years), n (%)**			
	65-74	18,882 (35.17)	
	≥75	34,808 (64.83)	
**Admitted from, n (%)**			
	Home	36,792 (68.52)	
	Health care setting^b^	16,898 (31.47)	
**Discharged to, n (%)**			
	Home	33,738 (62.83)	
	Health care settings	17,306 (32.23)	
	Died in hospital	2646 (4.92)	
**Length of stay**			
	Mean (SD)	12.26 (16.5)	
	Median (IQR)	8 (4-15)	
	Minimum-maximum	1-1316	

^a^CH: Switzerland.

^b^Nursing homes, other hospitals, and socioeducational and long-term psychiatric rehabilitation settings.

**Table 2:** Distributions of the e-Frail-CH^a^ instrument’s dimensions after cluster analysis (n=46,743 at admission, n=47,361 at discharge).

Dimensions and quality of cluster^b^		Score	Admission conditions	Admissions distribution (%)	Discharge conditions	Discharge distribution (%)	Difference between hospital discharge and admission (*P* value; %)
**Cognition**							
	0.8	0	0+0+0+0	71.9	0+0+0+0	64.9	–7^c^
	Good	1	0+0+(1 + 1)	10	0+(1 or 1 or 1)	27.1	17.1
	N/A^d^	2	Other	18	Other	8	–10
**General health status (*ICD-10^e^*/CHOP^f^)**							
	Allocated manually	0	1 *ICD-10* or 1 CHOP	1 *ICD-10* or 1 CHOP	1 *ICD-10* or 1 CHOP	2.4	N/A
	N/A	1	2 *ICD-10* or 2 CHOP	2 *ICD-10* or 2 CHOP	2 *ICD-10* or 2 CHOP	6.2	N/A
	N/A	2	>2 *ICD-10* or >2 CHOP	>2 *ICD-10* or >2 CHOP	>2 *ICD-10* or >2 CHOP	91.4	N/A
**Functional Independence Measure**							
	0.7	0	0+0+0+0	49	0+0+0+0	47.3	–1.7 (ns)
	Good	1	(0 or 1) + 1+0+0	31.4	One or (1 or 2)+0+0	35.1	3.7
	N/A	2	Other	19.7	Other	17.6	–2.1
**Social support**							
	0.5	0	Married and living at home	Married and living at home	Married and living at home	29.9	N/A
	Fair to good	1	Single or divorced, or hospital and living at home	Single or divorced, or hospital and living at home	Single or divorced, or hospital and living at home	32.8	N/A
	N/A	2	Other	Other	Other	37.3	N/A
**Medication**							
	Allocated manually	0	<5 medications	75.7	75.7	32.5	–43.2^c^
	N/A	1	≥5 medications	24.3	24.3	67.5	43.2
**Mood**							
	Allocated manually	0	No mood disorders and no exhaustion	75.7	75.7	77	1.3^c^
	N/A	1	Mood disorders or exhaustion in *ICD-10*	24.3	24.3	23	–1.3
**Continence**							
	Allocated manually	0	0+0	84.6	0+0	83.4	–1.2^c^
	N/A	1	Other	15.4	Other	16.6	1.2
**Self-reported performance**							
	0.6	0	0+0+0+0+0	45.9	0+0+0+0+0	44.2	–1.7^c^
	Good	1	Other	54.1	Other	55.8	1.7

^a^CH: Switzerland.

^b^Divisive coefficients.

^c^*P*<.001; ns=nonsignificant, for Wilcoxon signed rank test, 2-tailed.

^d^N/A: not applicable.

^e^*ICD-10*: *International Classification of Diseases, Tenth Revision*.

^f^CHOP: Swiss classification of surgical procedure.

### Variable Selection for the e-Frail-CH Measurement Instrument

Following a literature review on frailty instruments, the authors were inspired by the selection of clinical data and variables reported in the dimensions covered by the Edmonton Frail Scale (EFS; Table 2) [29]. The EFS assessment tool comprises 10 items [29]. Considering this study’s retrospective nature, EHR data on the risk of falls replaced a physical assessment of mobility. The EFS has been validated against the comprehensive geriatric assessment tool and was shown to be reliable and feasible for routine use by nongeriatricians [30,31]. Rolfson et al [29] described scores ranging from 0 (not frail) to 17 (severely frail), with scores of 8 or above defining patients as frail [29]. We selected data associated with the clinical features of frailty measured by the EFS to compute a data-driven frailty measure [29]. Using this approach, the clinical variables of frailty were defined as (1) the presence of any of the selected codes (no=not present; yes=present) or (2) the proportion of the codes present (0=not present or good health; 1=sometimes present or moderate health; 2=always present or bad health).

We constructed our instrument using clinical information from the following 9 dimensions of frailty: cognition (0=no errors, 1=minor errors, or 2=other errors), general health status (0=good health, 1=1-2 hospital visits, or 2=more than 2 hospital visits), functional independence (0=0-1 activities of daily living [ADLs] requiring help, 1=2-4 ADLs require help, or 2=5-8 ADLs require help), informal social support (0=always present, 1=sometimes present, or 2=never present), medication use (0=no polypharmacy or adherence problems, or 1=polypharmacy or adherence problems), nutrition (0=no weight loss or 1=substantial weight loss of >10%), mood (0=no depression or 1=depression), continence (0=no urine incontinence or 1=urine incontinence), and self-supported performance (0=able to do heavy work, walk upstairs, or walk 1 km; or 1=not able to do heavy work, walk upstairs, or walk 1 km). Scores of 0-7 were considered “not frail.” Scores of 8-9, 10-11, and 12-17 were considered “mild frailty,” “moderate frailty,” and “severe frailty,” respectively [32].

### Construction of the Data-Driven Frailty Measure Based on the EHR

The inpatient EHR dataset extracted included each older adult hospitalization between January 1, 2015, and December 31, 2017—3 years selected based on the availability of systematic, well-coded patient data. In addition, variables were selected according to this study’s aim and as closely as possible to the EFS, which we used as a reference instrument to create the e-Frail-CH for this study.

### Description of the Data

We selected the 8 dimensions of frailty listed in Table 2 (column 1), their indicators (column 2), their aggregated response categories (column 3), and their corresponding codes (column 4). We used them to construct the clusters for each dimension. Columns 5 and 6 present each response category’s distribution (as a percentage) within its respective dimension at hospital admission and discharge (when available). Note that the dimension of nutrition was not included in the e-Frail-CH instrument due to high numbers of missing values (92.7% missing at admission and 83.8% missing at discharge). Overall, patients at discharge presented with higher levels of dependency than at admission. In the dimension of functional independence, patients’ ability to self-care for their upper body increased (62.11% to 63.42%) between admission and discharge. The same result was noted for mood, with more participants feeling exhaustion at admission than at discharge (18.6% fell to 17.39%), and for an indicator of continence, with more urine drainage devices used at admission than at discharge (9.59% improved to 9.39%). Finally, for the dimension of cognition, the evaluation of patients’ states of consciousness found that 97.38% were fully alert at admission, 2.39% were in a state of drowsiness or stupor, and 0.19% were comatose. These proportions were 95.49%, 3.49%, and 0.99% at discharge, respectively. Table 2 presents all these data extracted from the Valais Hospitals’ register and used to construct the e-Frail-CH instrument. Almost all the differences between admission and discharge shown in Table 2 (last column) were highly significant (*P*<.001). However, the distributions of only 2 indicators were not significantly different, namely upper body self-care skills and having a urine drainage device.

### Data Analysis

In total, 2 experienced data managers (BW and HV) inspected the EHR dataset for extraction errors, missing values, and data consistency. Missing values were not replaced. Data were then imported into SPSS software (version 29, IBM Corp), for analysis. The e-Frail-CH instrument was constructed using hierarchical cluster analysis. This statistical technique involves grouping patients with similar characteristics in the dataset, such as similar diagnosis codes, clinical features, or self-reported performances. After examining the characteristics of each group derived from the hierarchical cluster analysis, a group with 6 or more diagnoses indicative of frailty was identified as the “frailty group.” For most of the dimensions of frailty, such as cognition, functional status, social support, and self-reported performance, we created clusters of variables based on an exploratory algorithm that uses the SPSS TwoStep Cluster Analysis procedure—a flexible, adaptable tool for clustering data. This exploratory procedure is designed to reveal natural groupings (or clusters) within a dataset that would not otherwise be apparent. Its algorithm has several advantages over traditional clustering techniques: (1) it can create clusters based on both categorical and continuous variables, (2) the number of clusters can be selected automatically or manually, and (3) it can analyze large data files efficiently. The TwoStep Cluster Analysis procedure handles categorical and continuous variables using a likelihood distance measure that assumes that variables in the cluster model are independent. The number of clusters to be formed is based on the Schwarz-Bayesian information criterion [33]. The higher the coefficient, the better the corresponding algorithm, as this coefficient is akin to a correlation coefficient [34]. Based on a Kolmogorov-Smirnov analysis, all the data included in the cluster analysis showed normal distributions, and the e-Frail-CH scores at admission and discharge were compared using a 2-tailed paired *t* test.

### SPSS TwoStep Cluster Analysis Algorithm for the e-Frail-CH Instrument

The 2-step procedure’s first step is constructing preclusters to reduce the matrix size and the distances between all the possible pairs of data points. Preclusters are simply clusters of the original cases that are used in place of the raw data in the hierarchical clustering. As a case is read, the algorithm decides, based on a distance measurement, whether the current case should be merged with a previously formed precluster or start a new precluster. In the second step, SPSS uses the standard hierarchical clustering algorithm on the preclusters. This refines the initial estimate by finding the most significant change in distance between the 2 closest clusters in each hierarchical clustering step. Forming clusters hierarchically allows us to explore a range of solutions with different numbers of clusters. At this point, the algorithm can propose a number of clusters, on an experimental basis, using the Schwarz-Bayesian information criterion, but the number of clusters can also be defined in advance. Our top-down, divisive approach groups all the observations into 1 cluster and then splits it recursively as we move down the hierarchy. The divisive coefficient varies from 0 (a poor coefficient) to 1 (a strong coefficient) across the observations [34,35]. Summing all the subcluster scores to create an overall frailty score made our strategy as close as possible to that used in the EFS framework and enabled us to count the score attributed to each of our different subdimensions. The scores of certain dimensions of frailty were defined directly in an affirmative manner, without clustering. This included the dimensions of general health status (defined by the number of *ICD-10* and CHOP [Swiss classification of surgical procedure] surgery intervention codes at discharge), medication (defined by the number of medicines prescribed at admission and discharge), mood (defined by the presence or absence of exhaustion or depression), and continence.

### Validity Analysis

We performed a validity analysis to explore the e-Frail-CH instrument’s sensitivity, specificity, and cut point accuracy between nonfrail and frail. The sensitivity analysis was constructed using hospital discharge data. We analyzed receiver operating characteristics (ROCs) to reveal the e-Frail-CH instrument’s trade-off between sensitivity and specificity [36]. The instrument was structurally validated using the multidimensional FIM as described by Naschitz et al [37] and Stuck et al [19,20,25,37,38]. The FIM instrument includes measures of independence in self-care, including sphincter control, transfers, locomotion, communication, and social cognition. It is an 18-item, 7-level, ordinal scale sensitive to the changes occurring during a comprehensive inpatient medical rehabilitation program. Its total score ranges from 18 (entirely dependent) to 126 points (completely independent), using the levels of assistance individuals need to grade their functional status between the extremes. The instrument can assess a patient’s level of disability or a change in their status in response to rehabilitation or a medical intervention. Our sample population’s scores were recorded as dependent patients (FIM scores of 18-53), patients with modified independence (54-107), and independent patients (scores from 108 to 126).

### Ethical Considerations

The Human Research Ethics Committee of the Canton of Vaud (2018-02196) gave its ethics approval, which enabled the research team to partner with the hospital’s data warehouse to access the appropriate dataset. Given the retrospective data source, obtaining consent from the patients concerned was impossible or posed disproportionate difficulties. This study respects the legal requirements for research projects involving data reuse without consent, as set out in Article 34 of Switzerland’s Human Research Act. As per Switzerland’s Federal Act on Data Protection, which is regulated at the federal and cantonal levels, patients’ sensitive personal data, such as their date of birth, address, educational level, and profession, were excluded from the dataset [39]. However, this study’s participants had signed a general consent form based on informed consent, in which they agreed that their data could be used for research purposes.

## Results

### e-Frail-CH Sample

Of the 53,690 hospitalization lines in the dataset, 46,743 and 47,361 met our inclusion criteria for computing 2-step hierarchical clustering for hospital admission and discharge, respectively. About 5.58% (2646/47,361) of the sample died during the hospitalization period selected and thus could not be considered at discharge.

### Constructing the e-Frail-CH Admission and Discharge Measurement Instrument

At the beginning of its construction, the e-Frail-CH measurement instrument’s 8 basic dimensions were selected based on the available EHR data that closely matched the EFS dimensions (Table 3, column 1). The selected dimensions’ clustering quality was computed and assessed using the SPSS TwoStep Cluster Analysis procedure (column 2). Column 3 presents each cluster-construction item’s score. In contrast, column 4 indicates the conditions used to attribute different variables to different hospital admission assessment categories, either in an exploratory way or manually, by using the order of the indicators in Table 2. Finally, column 5 presents the relative distributions (as percentages) of each dimension’s score at hospital admission. Columns 6 and 7 used the same information for hospital discharge.

The intermediate category for cognition indicators, coded 1, was attributed to patients admitted awake and conscious, displaying full capacity regarding temporal-spatial orientation but a limited ability to acquire knowledge or function in the ADL. The worst state of cognition, coded 2 at hospital admission, corresponded to any other indicator configuration. This clustering solution presented excellent summarizing quality, with a score of 0.8 on a scale from 0 to 1 (Table 2). The dimension of general health status (the same measure at hospital admission and discharge) was not clustered but defined in an affirmative manner based on the number of *ICD-10* and CHOP codes at hospital discharge only. The best health status, coded 0, was given to patients with just 1 *ICD-10* or CHOP code (2.39% of cases); the intermediate score, coded 1, was given to patients with 2 *ICD-10* or 2 CHOP codes (6.21% of cases), while the worst score, coded 2, was given to people with more than 2 *ICD-10* or CHOP codes (91.39% of cases; Table 3).

**Table 3:** Distribution of frailty scores at admission (n=46,743) and discharge (n=47,361).

e-Frail-CH^a^ score	Distribution at hospital admission, n (%)	Distribution at hospital discharge, n (%)	Relative difference in e-Frail-CH distribution from admission to discharge (%)
0 (no frail)	268 (0.57)	226 (0.47)	–0.1
1	1040 (2.22)	782 (1.65)	–0.5
2	5078 (10.86)	2784 (5.87)	–5
3	6871 (14.69)	6710 (14.16)	–0.5
4	7297 (15.61)	7350 (15.51)	–0.1
5	6546 (14)	6664 (14.07)	0.1
6	5438 (11.63)	5766 (12.17)	0.6
7	4373 (9.35)	5249 (11.08)	1.7
8	3436 (7.35)	3900 (8.23)	0.8
9	2670 (5.71)	2677 (5.65)	0
10	1878 (4.01)	2008 (4.23)	0.2
11	1488 (3.18)	2511 (5.30)	2.1
12 (severely frail)	360 (0.77)	734 (1.54)	0.7
Total	46,743 (100)	47,361 (100)	N/A^b^
Missing	6947	6329	N/A

^a^CH: Switzerland.

^b^N/A: not applicable.

### Cut Points Between the e-Frail-CH and FIM Scores at Hospital Discharge

Functionally unimpaired patients (with FIM scores ≥107) had optimal e-Frail-CH cutoff scores ≥6, with an excellent sensitivity cut point of 0.82, a specificity cut point of 0.88, and an area under the ROC curve of 0.85. Patients with the lowest FIM scores (18-53, n=272, 0.01%), indicating severe dependence, had optimal e-Frail-CH cutoff scores ≥9, with an excellent sensitivity cut point of 0.97, a specificity cut point of 0.88, and an area under the ROC curve of 0.92.

### Prevalence of Frailty Among Admitted and Discharged Patients

Based on the e-Frail-CH instrument and considering equivalent cutoffs based on the FIM score of 6, a total of 42.02% (n=19,643) of older adult patients were considered frail at admission, whereas 48.23% (n=22,845) were frail at discharge. e-Frail-CH scores are constructed by summing an individual’s scores for each dimension of frailty at hospital admission and discharge (Table 3). The theoretical score range is 0-12, with 0 meaning no frailty and 12 representing the highest state of frailty. The distribution approached normality for both scales with a slight positive skew. The mean hospital admission score (5.30, SD 2.59) was lower than at discharge (5.75, SD 2.65), indicating deterioration in most cases. The difference between the mean e-Frail-CH admission and discharge scores was significant: t_60,651_=0.45, 95% CI 0.44 to 0.48, *P*<.001).

### Association Between the e-Frail-CH and the FIM at Hospital Discharge

A strong negative association was found between e-Frail-CH scores and FIM scores at hospital discharge. Calculating a Spearman rank correlation (r_s_) of –0.844 (*P*<.001) showed that they were oriented in opposite directions.

## Discussion

### Principal Findings

Using routinely collected clinical and medical information, this research study developed a data-driven measurement instrument to compare older adult frailty at admission and discharge from Valais Hospitals’ multiple acute care sites. Frailty was measured using a cumulative approach, incorporating 8 dimensions of inpatients’ diagnostic, health, and clinical characteristics. These characteristics were very similar to those in prior studies measuring the prevalence of frailty based on accumulations of *ICD-10* diseases [28,40]. They were also similar to those in the studies by Hilmer et al [32] and Liang et al [28], which concluded that frailty appears to be a good predictor of adverse health outcomes and should be used as a health indicator in acute clinical practice to help formulate care and discharge plans and improve shared decision-making [32,41,42].

To transform frailty into a health indicator, we used hierarchical cluster analysis, a statistical technique that groups together individuals in a dataset whose measured characteristics are similar. These characteristics could be diagnosis codes or social, clinical, and medical data reported during a specified period [43,44]. However, hierarchical cluster analysis can require significant computing power for large datasets and yield different clusters in different datasets. Inspired by the components of EFS and Hilmer et al [32], this study tried to overcome the latter limitation by conducting its cluster analysis on a subset of a large hospital’s overall clinical dataset and developing a model to predict patients’ membership of different frailty groups [29]. Although our approach identified frail older adult individuals without requiring a dataset with a reference standard frailty measure, how the number of groups in the cluster analysis (anything other than a single, large frailty group) should be determined is open to interpretation. Frail older adult individuals will likely not all be classified into 1 group. For example, frail patients with cancer and frail patients with heart disease may be classified into different groups despite similar levels of frailty [40]. Our e-Frail-CH measurement instrument used information available within patients’ EHRs to identify their varying degrees of frailty. The 2-step hierarchical clustering strategy demonstrated robust results, with divisive coefficients between 0.5 and 0.8 fitting well with the results reported in similar studies [45,46]. e-Frail-CH’s high sensitivity, specificity, and areas under the ROC curve confirmed the instrument’s robustness. The EHR dataset included more than 45,000 valid hospitalizations or rehospitalizations used to construct the e-Frail-CH measurement instrument’s clusters. This should be considered an extremely big set of routinely collected data, especially when compared to the smaller samples used in the studies by Horn et al [47] and Pronk et al [47,48].

The summing of preclustered components enabled us to develop a clinical frailty score for older adults at acute hospital admission and discharge. Routinely identifying potentially frail older adult inpatients opens the way to targeted needs assessments and clinical alerts, better integrated care coordination, and coordinated planning to deliver necessary interventions during older adults’ health care trajectories and follow-up [18,20].

Our innovative approach aligns with a recent investigation in Spain by Orfila et al [40], who created and validated an electronic frailty score based on hospitalized patients’ clinical EHR data [40]. This study has the advantages of being inexpensive and transferable to other health care settings, especially in Switzerland. However, e-Frail-CH is not a clinical diagnostic tool—it is a population risk stratification tool that identifies groups of people likely to live with varying degrees of frailty. It cannot categorize specific individuals. Consequently, longitudinal assessments should take place to support the diagnosis when e-Frail-CH identifies an individual with potentially severe or moderate frailty. This study showed a 0.45-point mean increase in the frailty score between hospital admission and discharge. Although this value is essential for research purposes, the magnitude of such an e-Frail-CH score change might have clinical implications for individuals.

The e-Frail-CH instrument can be used as a clinical alert instrument in longitudinal retrospective studies and tested as a continuous variable. However, although it is suitable for acute care settings, the e-Frail-CH instrument’s screening effectiveness should be tested and compared against instruments screening for frailty in specific diseases or among preoperative patients. On the other hand, the EFS, the inspiration for our frailty scale, has recognized good psychometric properties [29,49,50]. The e-Frail-CH measurement instrument provides new insights into the potential for data-driven frailty assessments and comparisons of patient frailty during acute hospital trajectories [51,52]. Frailty remains complex, often undetected or confounded with other geriatric conditions or comorbidities [53]. To respond positively to frailty and its challenges, acute health care institutions need to be prepared for it: they should attempt tertiary preventive measures to take care of people exhibiting frailty and, where possible, report frailty scores for optimal hospital care planning. This could lead to targeted assessments and person-centered care plans being developed as the condition progresses to severe frailty. Alternatively, e-Frail-CH could guide optimized integrated care planning to help reverse emerging frailty among at-risk older adults [47,54]. Other investigations on data-driven electronic frailty indicators [8,39,55] corroborate our ideas about the e-Frail-CH measurement instrument. However, our investigation of frailty at acute hospital admission and discharge presents the added value of allowing an evaluation of changing frailty scores between these 2 critical points in a care trajectory. Additionally, e-Frail-CH enables patients who are frail to be distinguished from fit ones. Recent clinical guidelines recommend that inpatients be screened for frailty using a validated clinical alert instrument appropriate to the clinical setting, whether it is data-driven or not [53].

### Strengths and Limitations

One of this study’s main strengths was its innovative use of hospital register data-driven frailty detection or clinical alert indicator in acute care. To the best of our knowledge, few studies have investigated frailty using hospital registers. Another strength was using an approach that hierarchically clustered existing data to show frailty at hospital admission and discharge. Using an existing large-scale dataset and coding from patients’ EHRs was a crucial advantage because we did not have to solicit busy clinicians or patients who are frail and already weakened by multiple health conditions.

Nevertheless, this study had some limitations, notably its retrospective design and use of routinely collected hospital admission and discharge data. However, this could be considered a strength based on the clinical relevance of data in real-life hospital trajectories. As a result, we were unable to include data on patient nutrition due to that variable’s absence in the database for the selected period; nutrition is an essential dimension in assessing frailty [29]. Additionally, we could not control for potential data assessment errors made by health care staff during admission and discharge hospitalization. We could not suggest that our CH-e-frailty indicator was in a causal relationship with hospitalization trajectories, merely that it might serve as a clinical alert that could be communicated to other health care professionals (such as in a discharge letter) taking over responsibilities for older inpatients. Without a unique, encrypted patient identifier, we were unable to track inpatients who might have undergone multiple hospitalizations from 2015 to 2017, including some who died during their stay. The absence of data on patients’ functional status preadmission meant that we could not assess changes to that status during hospitalization or the influence of improving or deteriorating functional and cognitive impairments. Furthermore, using the FIM as an instrument for making comparisons should be carried out cautiously as it does not measure frailty directly but rather the level of disability. Further research should be conducted to reveal the profiles of older adult inpatients at risk of frailty. This means we must be careful about the external validity of our results and their interpretations. Finally, our findings should be considered with care because it was impossible to detect rehospitalizations, which may be a confounding factor in measuring frailty during hospital trajectories.

### Conclusion

The e-Frail-CH measurement instrument developed in this study could be applied widely across Switzerland to make the most of the routinely collected data on older adult inpatients in its acute care hospitals. This study demonstrated that patients’ EHRs contain easily extractable data that can be used to identify frail inpatients and to compare measures of frailty at admission and discharge. Furthermore, by using the e-Frail-CH measurement instrument, frailty could easily be identified using broader sociodemographic data and patients’ EHRs. Introducing a routine estimation of potential frailty among older adults via automated e-Frail-CH measurements could be a valuable way of identifying at-risk patients or clinical indicator and managing their frailty along their health trajectories. Further research could be carried out by using the e-Frail-CH measurement instrument score as both a long-term outcome and a patient-reported outcome [56].

## Appendix

Multimedia Appendix 1 Description and distribution of the variables included in the e-Frail-CH frailty indicator. CH: Switzerland.

## Abbreviations

ADL: activity of daily living
CH: Switzerland
CHOP: Swiss classification of surgical procedure
EFS: Edmonton Frail Scale
EHR: electronic health record
FIM: Functional Independence Measure
*ICD-10*: *International Classification of Diseases, Tenth Revision*
*ICD-11*: *International Classification of Diseases, Eleventh Revision*
ROC: receiver operating characteristic

## Data Availability

As part of the Data Use Agreement, the authors are not allowed to provide raw data. Upon a reasonable request, the corresponding author will provide the statistical programming code used to generate results.
