# Supplementary material for: Using Existing Clinical Data to Measure Older Adult Inpatients’ Frailty at Admission and Discharge: Hospital Patient Register Study
Source: JMIR Aging. 2024 Oct 28;7:e54839. doi: 10.2196/54839 (PMC11555450; doi:10.2196/54839)
Supplement: Multimedia Appendix 1 [file aging_v7i1e54839_app1.docx]

**Table S1**. Description and distribution of the variables included in the e-Frail-CH frailty indicator.

| Dimensions and indicators | | Categories | Codes | Distribution at hospital admission (n=46,743, 87.06%) | Distribution at hospital discharge (n=47,361, 88.21%) | Difference between hospital discharge and admission (*P* value; %)^a^ |
| --- | --- | --- | --- | --- | --- | --- |
| **Cognition** | | | | | | |
|  | **Assessment of consciousness, wakefulness, or passive attention** | | | | | |
|  |  | Awake | 0 | 97.4 | 95.5 | –1.9***a |
|  |  | Drowsy and stuporous | 1 | 2.4 | 3.5 | 1.1 |
|  |  | Comatose | 2 | 0.2 | 1.0 | 0.8 |
|  | **Assessment of temporospatial, place, or person orientation** | | | | | |
|  |  | 3 capacities present | 0 | 83.2 | 78.8 | –4.4*** a |
|  |  | 1 or 2 capacities present | 1 | 12.9 | 16.1 | 3.2 |
|  |  | 0 capacities or not assessable | 2 | 3.8 | 5.1 | 1.3 |
|  | **Capacity to acquire knowledge (assimilation of information)** | | | | | |
|  |  | Full capacity | 0 | 79.2 | 71.2 | –8*** a |
|  |  | Slightly and severely restricted | 1 | 18.5 | 24 | 5.5 |
|  |  | Disability | 2 | 2.3 | 4.8 | 2.5 |
|  | **Life skills, ability to act, or react to demands** | | | | | |
|  |  | Full capacity | 0 | 79 | 72.3 | –6.7***a |
|  |  | Little and highly restricted | 1 | 18 | 23.5 | 5.5 |
|  |  | Nonexistent or not assessable | 2 | 3 | 4.2 | 1.2 |
| **General health** | | | | | | |
|  | *ICD-10^b^* diagnoses | Number of *ICD-10*, mean (SD) | N/A | N/A | 6.7 (3) | N/A |
|  | CHOP^c^ diagnoses | Number of CHOP, mean (SD) | N/A | N/A | 1.67 (2.21) | N/A |
| **Functional independence** | | | | | | |
|  | **Self-care skills or upper body care** | | | | | |
|  |  | Full capacity | 0 | 62.1 | 63.4 | 1.3 (ns)a |
|  |  | Slightly and severely restricted | 1 | 33.7 | 30 | –3.7 |
|  |  | Disability | 2 | 4.2 | 6.6 | 2.4 |
|  | **Self-care skills or lower body care** | | | | | |
|  |  | Full capacity | 0 | 51.5 | 50.9 | –0.6***a |
|  |  | Slightly and severely restricted | 1 | 37.6 | 34.8 | –2.8 |
|  |  | Disability | 2 | 10.9 | 14.3 | 3.4 |
|  | **Capacity in the gestures allowing eating** | | | | | |
|  |  | Full capacity | 0 | 86.8 | 84.1 | –2.7***a |
|  |  | Slightly and severely restricted | 1 | 10.2 | 11.6 | 1.4 |
|  |  | Disability | 2 | 3.1 | 4.4 | 1.3 |
|  | **Capacity in the gestures allowing drinking** | | | | | |
|  |  | Full capacity | 0 | 92.4 | 89.5 | –2.9***a |
|  |  | Slightly and severely restricted | 1 | 5.6 | 7.2 | 1.6 |
|  |  | Disability | 2 | 2 | 3.4 | 1.4 |
| **Social support** | | | | | | |
|  | **Marital status** | | | | | |
|  |  | Married | 0 | 53.5 | N/A | N/A |
|  |  | Single | 1 | 7.8 | N/A | N/A |
|  |  | Divorced or separated | 2 | 38.7 | N/A | N/A |
|  | **Origin** | | | | | |
|  |  | Home | 0 | 71.4 | N/A | N/A |
|  |  | Hospital | 1 | 26.8 | N/A | N/A |
|  |  | Long-term care facilities | 2 | 1.9 | N/A | N/A |
|  | **Destination** | | | | | |
|  |  | Home | 0 | N/A | 62.9 | N/A |
|  |  | Hospital | 1 | N/A | 24.7 | N/A |
|  |  | Long-term care facilities | 2 | N/A | 8.4 | N/A |
|  |  | Death | 2 | N/A | 4 | N/A |
| **Medication** | | | | | | |
|  | **Medication** | | | | | |
|  |  | <5 medications | 0 | 75.7 | 32.5 | –43.2***b |
|  |  | ≥5 medications | 1 | 24.3 | 67.5 | 43.2 |
| **Mood** | | | | | | |
|  | **Mood disorders** | | | | | |
|  |  | No F300-F399 in *ICD-10* | 0 | N/A | 93.8 | N/A |
|  |  | One or more F300-F399 codes in *ICD-10* | 1 | N/A | 6.2 | N/A |
|  | **Feelings of exhaustion** | | | | | |
|  |  | No exhaustion | 0 | 81.4 | 82.6 | 1.2***b |
|  |  | Exhaustion | 1 | 18.6 | 17.4 | –1.2 |
| **Continence** | | | | | | |
|  | **Miction** | | | | | |
|  |  | Miction control | 0 | 86.2 | 84.3 | –1.9***b |
|  |  | No miction control | 1 | 13.8 | 15.7 | 1.9 |
|  | **Urine drainage device** | | | | | |
|  |  | No | 0 | 90.4 | 90.6 | 0.2 (ns)b |
|  |  | Yes | 1 | 9.6 | 9.4 | –0.2 |
| **Self-reported performance** | | | | | | |
|  | **Mobility—movement** | | | | | |
|  |  | Total mobility or not very restricted | 0 | 76.5 | 82.6 | 6.1***b |
|  |  | Severely restricted mobility or unable to move | 1 | 23.5 | 17.4 | –6.1 |
|  | **Mobility—change of position** | | | | | |
|  |  | Total mobility or not very restricted | 0 | 85.9 | 87.6 | 1.7***b |
|  |  | Severely restricted mobility or unable to move | 1 | 14.1 | 12.4 | –1.7 |
|  | **Altered gait** | | | | | |
|  |  | No | 0 | 56.9 | 54.6 | 2.3***b |
|  |  | Yes or not accessible | 1 | 43.1 | 45.4 | 2.3 |
|  | **Balance disorder** | | | | | |
|  |  | No | 0 | 63.5 | 65.4 | 1.9***b |
|  |  | Yes or not accessible | 1 | 36.5 | 34.6 | –1.9 |
|  | **Fall before hospitalization** | | | | | |
|  |  | No | 0 | 77.8 | 73.4 | 4.4***b |
|  |  | Yes or not accessible | 1 | 22.2 | 26.6 | 4.4 |

^a^****P*<.001, ***P*<.01, **P*<.05, ns=nonsignificant, for the Wilcoxon signed rank (^a^) test and the two-tailed paired *t* test, (^b^) respectively, N/A = not applicable.

^b^*ICD-10*: *International Classification of Diseases, Tenth Revision.*

^c^CHOP: Swiss classification of surgical procedures.
